# Flipped classroom model versus conventional teaching method: effects on nursing students’ self-directed learning readiness in a research methodology course

**DOI:** 10.11604/pamj.2024.47.70.38359

**Published:** 2024-02-19

**Authors:** Paulina Chigwara Chikeme, Chikaodili Ndidiamaka Ihudiebube-Splendor, Ngozi Phoebe Ogbonnaya, Chisom Joy Mbadugha, Laurentia Onyinye Elodi

**Affiliations:** 1 Department of Nursing Sciences, Faculty of Health Sciences and Technology, University of Nigeria, Enugu Campus, Enugu, Nigeria,; 2 Department of Educational Management and Policy, Chukwuemeka Odimegwu Ojukwu University, Igbariam, Nigeria

**Keywords:** Teaching methods, self-directed learning readiness, flipped classroom model, conventional teaching method, nursing

## Abstract

**Introduction:**

the effectiveness of the flipped classroom model (FCM) method for building self-directed learning readiness (SDLR) in a research methodology course has not yet been scientifically researched. This study aims to assess the effect of FCM on SDLR among Nigerian nursing students enrolled in a research methodology course.

**Methods:**

sixty-four 400-level nursing students from two government-owned universities in Southeast Nigeria were recruited for this quasi-experimental study. They were randomly divided into two groups, the experimental group, and the control group, and exposed to FCM and conventional teaching methods (CTM) throughout the months of April and July 2021. Before and after the intervention, the validated self-directed learning readiness scale (SDLRS) was used to gather data, and a structured questionnaire was used to collect demographic data.

**Results:**

the SDLR was high if the SDLRS mean score was ≥3.75. Pre-test results from SDLR showed overall scores of 3.99 ± 0.39 and 3.95 ± 0.35 for CTM and FCM, respectively, while post-test results showed overall scores of 3.84 ± 0.77 for CTM and 4.01 ± 0.81 for FCM. The mean scores between the pre-and post-tests were statistically different (p=0.030).

**Conclusion:**

the FCM had a positive effect on Nigerian nursing undergraduates' SDLR and should be encouraged as it provides a viable alternative to the traditional teaching method.

## Introduction

Research methodology is a mandatory course in the nursing education curriculum. Knowledge of research not only raises nursing care standards and quality, but it can also help nurses advance their careers [1]. The conventional teaching method and flipped classroom model approaches are used to teach the research process. The conventional teaching method (CTM) is a teacher-centered method of instruction where the instructor stands in front of a class of students and offers knowledge for them to absorb [2,3]. Despite the fact that the usage is noted in small groups [3,4]. The conventional teaching method is the best strategy when working with a large group when the facts or situations are ambiguous or contradictory. When oral communication of a material is the best way to understand it, the lecture method works best. Complex concepts can be organized, clarified, and explained by instructors during conventional teaching. More crucially, the teaching is entirely under the instructor's control. When teaching certain facts and fundamental skills, the conventional teaching technique is crucial since it makes space for the local and direct presentation of factual knowledge [5].

Despite the benefits, the CTM has its flaws because it is a one-way process with minimal discussion, probing, or hands-on practice, resulting in students having more superficial knowledge than other teaching methods [5]. Students learn less utilizing CTM than they do while using FCM because it places more emphasis on information than on the learners [6,7]. This might be explained by the CTM's directive approach, which tells students what to do rather than encouraging them to explore on their own [8]. The inference is that the instructor´s knowledge and experience are the only ones available to the students, which is detrimental to the development of higher-order thinking skills [9], has the ability to inhibit students' creativity, and encourages rote learning. Because of the shortcomings of CTM, academics started looking for alternatives, which gave rise to the flipped classroom model (FCM) method.

The flipped classroom model (FCM) method is described as the student-centered method of teaching that enables students to absorb theoretical material on their own at home and apply what they learn in school [10]. It entails moving material delivery outside of formal class time (through extensive notes, video-recorded lectures, and other appropriate means) and using formal class time for students to engage in collaborative activities related to the material [11,12]. The method encourages students to participate more actively in interactive activities inside the classroom while transferring more traditional activities outside of the classroom. Therefore, one of the many benefits is that it is aligned with Bloom's taxonomy [13]. Rather than traditional classroom instruction that requires lower levels of thinking skills, the flipped model allows students to develop higher-order thinking skills during class time with teacher guidance and peer support [14]. In the conventional teaching approach when the teacher is not present, the students complete the task that challenges them on their own [15]. The flipped classroom method is essential for students' improvement because it enables them to learn fundamental information from outside activities, readings, and other materials while working on difficult and higher-order cognitive tasks in the classroom [16]. Effective use of the flipped classroom model requires the acquisition of certain skills. Self-directed learning (SDL) skill is one such skill. Self-directed learning is a process in which a person analyzes their learning needs, formulates learning goals, discovers human and material resources for learning, chooses and executes appropriate learning strategies, and evaluates learning outcomes with or without the help of others [17]. In the context of nursing education, SDL is defined as learning in freedom when the learner assumes the main responsibility for predetermining what is to be done when it is to be done, and how and evaluating the effort [18]. The primary idea is that students are in charge of their own education beyond what is provided by an outside source (e.g., faculty member, the curriculum). More so with SDL, the student takes charge by formulating learning goals, choosing tests that offer feedback, and locating resources to help them reach their goals. Students take responsibility for any behaviours that were previously prescribed by the instructor and are now free to encourage learning by taking control of their education [19,20]. Thus, this raises students' self-directed learning readiness (SDLR), which is necessary to improve their exam performance.

Consequently, SDLR gives the student a sense of mastery, independence, and purpose in the learning process. The self-directed learning readiness scale (SDLRS), which consists of self-control, learning desire, and self-management subscales, can be used to assess the SDLR. In addition to successfully enhancing students' emotional and motor skills, the advantages can be linked to more knowledge [21]. The results of studies showed studies that students exposed to SDL performed better compared to the conventional teaching group in terms of performance [22]. Also, in a related study assessing clinical skills, knowledge, and attitudes [23]. Murad *et al*. asserted that the SDL approach was more efficient and superior to CTMs [23].

However, studies have revealed that the performance of university-based nursing students in Southeast Nigeria who are taught research methodology utilizing CTM is declining [24,25]. The instructional strategies employed are to blame for this substandard result. The requirement for this study results from the necessity of testing a different teaching strategy. The results of this study are anticipated to add to the body of knowledge, as well as shed light on the extent to which nursing undergraduates possess SDLR, as well as the best instructional strategies for fostering SDLR. Additionally, it will affect the success rate and the strategies used to teach students. Therefore, the specific goals of this study were to ascertain the SDLR levels of nursing undergraduates in the chosen department of nursing sciences prior to exposure to treatment, evaluate the levels of SDLR following exposure to treatment, and determine whether there was a statistically significant difference between the SDLR levels of nursing undergraduates exposed to FCM and those exposed to CTM. Therefore, the purpose of this study is to evaluate the effect of FCM on students offering the research methodology course in universities in Southeast Nigeria.

## Methods

**Study design and participants:** a quasi-experimental study involving two classes of nursing undergraduates at the 400-level from two government-owned postsecondary institutions in Southeast Nigeria. Class B received instruction based on conventional teaching approaches (control group), while class A received treatment using the flipped classroom model (FCM). A total of 64 students were enrolled in the study, 32 of whom were in each class. The two classes had similar overall Cumulative Grade Point Averages (CGPAs) over the preceding three years, but there were individual grade level discrepancies. Nevertheless, the distribution of high achievers and low achievers on the achievement exam was relatively comparable between the two classes. According to their transcripts from the preceding three years, class A students with a CGPA of ≥2 were considered high achievers, while those with a CGPA of < 2 were considered low achievers. The distribution of test scores for the two classes, in this case, was quite similar. Due to the small number of males in the study, all males who met the inclusion criteria took part (having complete results), whereas the females were chosen at random. The assessment was based on the effects of the teaching methods; therefore, the lack of males was made up for by the projected number of girls with the same academic competence.

**Data collection:** Fisher *et al*. created the 40-item self-directed learning readiness scale (SDLRS), which was requested and utilized in this investigation [26]. The 40 items were divided into three domains: self-management (13 items), control of one's learning (15 items), and desire for learning (12 items) (13 items). Higher scores indicated higher patterns of SDL, and participants were asked to choose from a 5-point Likert scale ranging from 1 (strongly disagree) to 5 (strongly agree). Items 3, 11, 20, and 40 were negatively phrased and their scores were reversed when calculating the total and subscale scores to prevent response set bias. The internal reliability coefficient in the Fisher *et al*. study was 0.857 for the sub-dimension of self-management, 0.843 for a desire for learning, 0.830 for self-control, and the total SDLR was 0.93 [26]. In this study, the reliability coefficients were subscale 1=0.756 (self-management), subscale 2=0.730 (desire for learning), subscale 3=0.908 (self-control), and overall =0.945. The difference between pretest and posttest scores on students' SDL abilities reflects the efficacy of the two instructional strategies on students' SDL.

The self-developed demographic characteristics questionnaire comprised four items which included the name of the institution, age, gender, and marital status. The Institutional Review Board of the University of Nigeria Teaching Hospital Enugu, Nigeria, granted the researcher ethical approval before delivering the test (NHREC/05/01/2008B-FWA00002458-IRB00002323). Participants provided written informed consent, and each participant's assessment tools were given an untraceable number in order to protect their privacy.

### Experimental procedures

**Flipped classroom model:** the student's past three years of transcripts were used to determine the experimental and control groups based on the necessary ethical permission. Similar representations of high and low academic achievers were seen in both groups (FCM and CTM). The self-directed learning readiness scale was administered to the experimental and control groups prior to the procedure (SDLRS). In the experimental group, the flipped classroom paradigm was used as planned to deliver the eighth-week research methodology package. Every Monday at 6 p.m., prior to the Wednesday in-class, the researcher created the research methodology material in audio PowerPoint format and distributed it via a platform established by the group. Students read the texts and listened to the audio versions of the texts at home, followed by classroom engagement, which is more student-focused. The teacher guides the activities being carried out, encourages active engagement from the students, and works as a facilitator and supporter of learning. Every week when the class met again, the students were encouraged to express their worries and uncertainties and to ask questions based on their prior experiences in order to get an explanation. Following the FCM sessions, quizzes based on the lesson objectives were used to evaluate the group. One week before the final exam, the researcher gave the students the SDLRS to assess the effect of the teaching methods on their SDLR (post-test).

**Conventional teaching method:** a lecture-based teaching strategy was employed in the conventional classroom which is a common practice in Nigeria's higher institutions. The research methodology course material was similar to that for FCM. The teacher assigns readings to the group for the upcoming lecture during each weekly class meeting, along with related reference books. A total time of 1.30 hours of lecture and 30 minutes of interaction was spent on teaching each day. This timeframe is comparable to that of FCM group weekly group interactive learning sessions. Additionally, lecture notes were sent out to the students following each weekly classroom lesson; these lecture notes gave the students pertinent information for independent study and review. In contrast to the FCM group, which got an online audio PowerPoint lesson through the WhatsApp platform prior to in-class teacher-student interaction, CTM students solely received lecture-based education through the projected PowerPoint slides. Throughout the 8 weeks, the group received instruction every Monday.

**Data analysis:** the Statistical Package for the Social Sciences (SPSS) version 25 and Microsoft Excel were used to conduct the data analysis. The scaled components were analyzed using descriptive statistics of the means and standard deviations. Any response < 3.75 was seen as having a low SDLR, whereas ≥3.75 indicated a high SDLR. The difference between the pre and post-test scores of FCM and CTM students was examined using an independent sample t-test with a 0.05 level of significance.

## Results

In Table 1, the overall rating of self-management was fairly high in the CTM group (3.81 ± 0.55) and low in the FCM (3.53 ± 0.60). The desire for learning was high in the 2 groups (CTM (4.19 ± 0.50), flipped (4.21 ± 0.36)) while self-control was fairly high in the CTM (3.98 ± 0.44) and high in the FCM group (4.09 ± 0.40). The overall SDLR was fairly high in the 2 groups (CTM (3.99 ± 0.39) and flipped (3.95 ± 0.35)).

**Table 1:** means and standard deviations of pre-test self-directed learning readiness in students exposed to FCM and CTM-based learning

Item	CTM (n=32)	FCM (n=32)
	**Mean ± SD**	**Mean ± SD**
Self-management	*3.81 ± 0.55	*3.53 ± 0.60
Desire for learning	+4.19 ± 0.50	+4.21 ± 0.36
Self-control	+3.98 ± 0.44	+4.09 ± 0.40
Self-directed learning readiness	*3.99 ± 0.39	+3.95 ± 0.35

* skills rated fairly high; + skills rated high; domain and sub-domain means were used instead of totals; FCM: flipped classroom model; CTM: conventional teaching methods

Table 2, shows the overall rating of self-management was fairly high in the CTM (3.75 ± 0.64) and FCM groups (3.86 ± 0.63). The desire for learning was high in the 2 groups (CTM (4.00 ± 0.71), FCM (4.69 ± 0.42)) while self-control was fairly high in the CTM (4.01 ± 0.40) and high in the FCM group (4.15 ± 0.52). The overall SDL readiness was fairly high in the CTM (3.84 ± 0.77), and high in the FCM (4.01 ± 0.81).

**Table 2:** means and standard deviations of post-test self-directed learning readiness in students exposed to FCM and CTM-based learning

Variables	CTM (n=32)	FCM (n=32)
	**Mean ± SD**	**Mean ± SD**
Self-management	3.75 ± 0.64	*3.86 ± 0.63
Desire for learning	+4.00 ± 0.71	+4.69 ± 0.42
Self-control	+4.00 ± 0.40	+4.15 ± 0.52
Self-directed learning readiness	*3.84 ± 0.77	+4.01 ± 0.81

* Skills rated fairly high; + skills rated high; Domain & sub-domain means were used instead of totals; CTM: conventional teaching methods; FCM: flipped classroom model

Results in Table 3 showed that the students in the FCM group (MD = 0.18) improved more in their self-directed learning readiness than the students in the CTM group (MD = 0.08) following exposure to the intervention (p = 0.030). With the exception of self-control (MD = 0.07), the students in the FCM group improved more after the post-treatments in self-management (MD = 0.31), and desire for learning (0.16), than their counterparts in the CTM group. Although the p-values for the self-management, desire for learning, and self-control variables were all greater than 0.05, the difference in averages between the two groups was not statistically significant after the intervention.

**Table 3:** mean score difference in the self-directed learning readiness between pre-test and post-test of nursing undergraduates exposed to FCM and CTM group

Variables	FCM	CTM (n=32)	T	p-value
	Mean difference	Mean difference		
	FCM mean difference	CTM (n = 32) mean difference		
**Mean difference 1 (post 1 - pre)**				
Self-management	0.3069	0.1492	-1.882	0.072
Desire for learning	0.1642	0.0075	-1.910	0.069
Self-control	0.0720	0.0780	-0.130	0.898
Self-directed learning readiness	0.1760	0.0800	-2.209	0.030

FCM: flipped classroom model; CTM: conventional teaching methods

## Discussion

The purpose of this study was to determine how FCM and traditional teaching method (TTM) affected the SDLR of nursing undergraduates enrolled in a research methodology course in two selected tertiary institutions in Southeastern, Nigeria.

The results showed that aside from the self-management subscale, which had a low score for the FCM group, nursing undergraduates possessed the necessary levels of SDLR at the pre-test stage. This shows that greater effort and resources are required to improve autonomous learning through the development of self-management skills. The CTM group had the best overall SDLR by a small margin. The study's encouraging findings are justified by the fact that the 400-level participants had past learning experiences over the preceding three years, which must have given them the ability to prioritize their learning and use the proper learning interventions. It is also possible that the difficulties and problems they encountered while working with patients during their clinical rotations gave them the information and skills, they needed to become more independent learners. This finding is in line with a study done in Australia among nursing students in which they performed poorly on the self-management subscale, better on the desire to learn, and at their best on self-control [27]. Additionally, at King Saud University, students scored lowest in the self-management domain and highest in the self-control subscale [28]. This finding contrasts with that of Williams *et al*. who claimed that while the desire for learning dimension had the highest mean score, the self-control and self-management dimensions had moderate scores [29]. The fact that just 64 students were enrolled in the current study while 259 participated in Williams *et al*. inquiry shows that the size of the sample size used in their investigation must have had an effect on the student´s scores on the self-control readiness scale. Contrarily various reports [30-33] noted that nursing students' probability to demonstrate SDLR was higher when exposed to the intervention than in the control group, the students were at their best with SDLR at the pretest stage without exposure to treatment. This finding could have been impacted by the sample size effect. In comparison to this study, earlier studies used more samples.

At the post-test stage, a significant improvement was seen in the mean SDLR score of students exposed to FCM compared to that of the CTM group. This contrasts with the pre-intervention stage outcome, which showed that the CTM group performed best. This indicates that there is a positive correlation between teaching strategies and the growth of SDL. This may be the case since flipped classroom instruction gives students the chance to interact with technology, putting them in charge of their own education. This makes the teaching process more engaging and student-centered, which may raise achievement levels. Along with working together in group projects and other hands-on activities, teachers also help students solve problems and participate in other activities that foster problem-solving abilities. All of these may result in greater increases in students' achievement scores. The above finding is consistent with earlier research [34] which found that the FCM was effective in enhancing urinary system knowledge and skill level, as well as nursing students' motivation and learning strategies. Additional studies [35] also found that using the flipping method, the students received significantly higher final marks in organic chemistry compared to those in the control group. The findings also corroborate with earlier research [36] that discovered that students who were exposed to flipped classrooms performed better academically than those who were not, as well as flipped learning can increase student engagement and achievement [37].

Results obtained by comparing the post-test and pre-test scores revealed that the FCM group's understanding of research methodologies, as evidenced by scores on the SDLR, was considerably higher than that of the CTM group except for the self-control subscale in which the CTM group was at best (p = 0.030). The fact remains that there are distractions with the online session of the flipped class. This means that while watching the lessons, it may have been difficult for the students to resist engaging in other activities like using Facebook, watching online football matches, or listening to music. Therefore, how to instill self-control readiness in learners while using this method should be an area of concern for instructors. However, this obtained result provides a more compelling argument for a relationship between the instructional approach and the growth of learner autonomy. It suggests optimizing the impact of SDLR; as opposed to the CTM method, an FCM-based instructional strategy must be developed. In light of this finding, the null hypothesis-according to which there is no statistically significant difference between the SDLR of students exposed to FCM and the group using the CTM-is debunked. The findings support the counterargument that FCM learning is more beneficial to students than CTMs. In light of these results, the study advises encouraging teachers to use FCM in the classroom. This finding is consistent with earlier research [38] that suggested flipped classroom settings might enhance self-directed learning. Similar to this, previous post-test results showed a decline in students' readiness for self-directed learning in the control group and an increase in the experimental group (control X=2,895 - experimental X=3,475) [39]. On the other hand, it goes against the findings of a study done in two distinct classes at Ahi Evran University, Faculty of Education which found there was no significant difference in self-directed learning preparedness of students exposed to FCM [36]. However, this gap may be attributed to the likelihood that the long-standing and well-liked lecture-based educational system affected the students who preferred passive learning. Another possibility may be that their instructors may have simplified the lecture-based teaching strategy.

**Limitations:** 1) The study should be broadened and extended to include more sample size and other levels of students; 2) the study sample was not gendered balanced; 70 (73%) of the participants were females, whereas 26(27%) were males, and the study lasted 8 weeks; 3) the research was conducted in universities located within a specific geopolitical region of Nigeria and does not necessarily reflect the views and opinions of other students in other regions of the country; 4) only a course in nursing (research methodology) was employed, other courses could as well be involved, and it could also be a full-semester intervention.

## Conclusion

The study examined the effects of FCM and CTMs on SDLR of nursing undergraduates in a research methodology class. The result showed that nursing students possessed the necessary levels of SDLR at the pre-test stage, except for the self-management subscale for the FCM group, the CTM group had a negligible overall SDLR. At the post-test stage students exposed to FCM showed a significant improvement in their overall mean of SDLR. Comparing the post-test - pre-test results, the FCM group´s SDLR was significantly higher than that of the CTM group (p = 0.030) except for the self-control subscale. Thus, FCM of instruction has a very good impact on students' SDLR. In order to teach nursing students to attain their academic and professional goals, nurse educators must be prepared with this method.

### What is known about this topic

- *Traditional teaching method (TTM) is still the most used and most preferred teaching method in tertiary institutions in Nigeria;*
- *The viability of FCM in helping students develop self-directed learning readiness (SDLR) in research methodology courses is not known;*
- *There is a paucity of literature in nursing education in Nigeria on the effectiveness of novel teaching methods in helping nursing students achieve SDLR*.

### What this study adds

- *The flipped classroom model (FCM) approach is gaining more popularity in most developed countries of the world;*
- *The flipped classroom model allows students to develop higher-order thinking skills during class time with teacher guidance and peer support;*
- *Since attitude is crucial for acquiring new knowledge, the FCM strategy should be used when teaching courses students to perform badly, such as research methodology*.
